# The association between adult IgA vasculitis and cancer: a prospective observational study

**DOI:** 10.3389/fmed.2025.1551772

**Published:** 2025-02-25

**Authors:** Alojzija Hočevar, Vesna Jurčič, Žiga Rotar

**Affiliations:** ^1^Department of Rheumatology, University Medical Centre Ljubljana, Ljubljana, Slovenia; ^2^Faculty of Medicine, University of Ljubljana, Ljubljana, Slovenia; ^3^Faculty of Medicine, Institute of Pathology, University of Ljubljana, Ljubljana, Slovenia

**Keywords:** adult IgA vasculitis, cancer, temporal relation, risk factor, urogenital cancers

## Abstract

**Objectives:**

Cancer has been reported as a potential trigger for IgA vasculitis (IgAV) in adults; however, data on this topic are scarce. The aim of our study was to examine the frequency and location of cancer in adults with IgAV.

**Methods:**

We included 295 IgAV patients diagnosed between January 2010 and June 2021 and followed at our secondary/tertiary rheumatology centre. Cancer episodes were recorded and classified into three groups according to their temporal relation to IgAV: (1) cancer diagnosed prior to IgAV; (2) cancer diagnosed concurrently with IgAV; and (3) cancer diagnosed during IgAV follow-up. The term IgAV associated with cancer (IgAV-CA) was used for IgAV cases presenting while cancer was active or diagnosed within a three-year period before or after the IgAV diagnosis.

**Results:**

Forty-seven IgAV patients (15.9%) developed a total of 56 different cancers. Of these, 43 cancers (76.8%) preceded the IgAV diagnosis, 3 cancers (5.3%) were diagnosed concurrently with IgAV, and 10 cancers (17.9%) were diagnosed during the IgAV follow-up. Twenty-two IgAV patients (7.5%) who developed a total of 24 cancers (42.9%) could be classified as IgAV-CA cases. The three most frequently diagnosed cancers in the IgAV-CA group were prostatic, renal and bladder cancer, together accounting for 50% of cancers. Age and smoking were associated with concurrent cancers, and arthritis represented a risk factor for cancer development during IgAV follow-up.

**Conclusion:**

IgAV cases associated with cancer represent 7% of adult IgAV cases. In elderly patients, careful examination of the urogenital tract is warranted when cancer is suspected.

## Highlights

- Seven per cent of adults with IgA vasculitis developed cancer within a three-year period before or after diagnosis of vasculitis.
- Urogenital tract cancers were the most prevalent in adult IgA vasculitis cases associated with cancer.
- In elderly patients with newly diagnosed IgA vasculitis cancer screening should be recommended as part of patient care.

## Introduction

Immunoglobulin A vasculitis (IgAV) is an immune complex-mediated small vessel leucocytoclastic vasculitis, typically affecting the skin, joints, gastrointestinal tract and kidneys (1). Although recognised as a classical children’s disease, IgAV is not infrequent in the adult population (2). Studies have shown that adults experience more severe disease compared to children (3), with a poorer long-term prognosis, particularly due to an increased risk for chronic kidney disease and worse survival (4, 5). Furthermore, potential triggers and characteristics of IgAV in adults are still less well explored compared to its paediatric cases. Genetic background and external triggers such as infections or medications, seem to be important at any age (6–8). Additionally, in adults, the coincidence of IgAV and cancer has been suggested, with a potential paraneoplastic background of the former (9). Overall, about 5% of vasculitis cases in adults are thought to be associated with cancer (10). Furthermore, a French study evaluating IgAV patients with nephritis reported cancer as the leading cause of death, accounting for 27% of all deaths (11). In our recent follow-up study, cancer was the cause of 12.5% of deaths (12).

Another French group found an even more severe IgAV phenotype in patients with associated cancer (13). Regarding the cancer location, a recent literature review, including just over 100 IgAV patients with cancer, found solid cancers in 67.6% and haematologic cancers in 32.4% of cases. Lung, genitourinary tract and gastrointestinal tract cancer represented the three most solid malignancies, accounting together for nearly 70% of cases, raising the hypothesis of aberrant IgA immunoglobulin synthesis in the mucosal tissue of cancer-affected organs. IgA plasma cell dyscrasia and lymphoproliferative disorder were the two most common hematologic neoplasms, together accounting for 97.1% of hematologic cancers (14). On the contrary a single center retrospective study from Japan did not observe any significant differences in organs affected by cancer (15). The Japanese study observed a unique pattern in the timing of cancer and IgAV. In their study, majority of patients developed IgAV after a cancer diagnosis and treatment (more than 83% of cases), while previously published cases showed that 75% of malignancies were diagnosed after IgAV episode (15).

The recent literature review detected temporal differences between solid and hematologic cancers. Solid cancers were in almost 80% diagnosed before or concurrently with IgAV [with a median time difference of 2 (IQR 0; 12) months], whereas hematologic cancers were diagnosed concurrently or after IgAV diagnosis (62.8%) with a median time difference was 3 months (IQR 0; 76 months) (14).

Given that current knowledge on IgAV cases with cancer stems mainly from reports of individual cases, small cases series and retrospective studies, the aim of our study was to prospectively determine the frequency and location of cancer. Additionally, we sought to identify potential differences between IgAV cases associated with cancer compared to non-cancer cases within an adult IgAV cohort under continuous observation.

## Methods

### Setting and patient selection

This observational study included consecutive adults (aged ≥18 years) diagnosed for the first time with IgAV between January 2010 and June 2021, who were followed prospectively at the Department of Rheumatology, University Medical Centre Ljubljana, Slovenia. All patients had histologically proven IgAV and fulfilled the EULAR/PRINTO/PRES classification criteria for IgAV at the time of diagnosis (16). Patients without histologically confirmed IgAV and IgAV patients diagnosed before 2010 were excluded from the study. There were no additional exclusion criteria applied.

### Baseline assessment of IgAV, treatment and follow-up

Patient demographics, comorbidities (including past cancer episodes) and baseline IgAV data were all recorded. Upon IgAV diagnosis, all patients underwent a detailed clinical, imaging and laboratory work-up for IgAV, as described previously (12), including at least chest X-ray, abdominal ultrasound or computed tomography, and tumour markers (CEA, CA 19-9, CA 15-3 in both sexes, PSA in males and CA 125 in females). Urine cytology was conducted only if there was a persistent extraglomerular haematuria or suspicious findings on urinary tract imaging. Based on the findings of the above investigations, further investigations for histopathological verification were performed if cancer was suspected, based on the suspected cancer location.

IgAV episode was considered severe in case of severe renal involvement (i.e., when nephrotic or nephritic syndrome with acute worsening of the kidney function developed) or in case of severe gastrointestinal involvement (defined as bloody diarrhea, ileus or bowel perforation).

Follow-up visits were scheduled at 2 to 4 weeks, 3, 6 and 12 months after IgAV diagnosis, and annually thereafter. Additional visits were arranged if IgAV worsened between scheduled appointments. At each visit, in addition to evaluating IgAV activity, the development of new comorbidities—including any newly diagnosed cancer or relapses—was recorded. For the purposes of this study, the censor date for data collection was set as 28 June 2024.

### Cancer episode classification

Cancer episodes were classified into three groups based on their temporal relation to IgAV diagnosis: (1) cancer diagnosed prior to IgAV; (2) cancer diagnosed concurrently with IgAV; and (3) cancer diagnosed during IgAV follow-up. Cases of non-melanoma skin cancer were excluded from the analysis for the purpose of this study.

The term IgAV cancer-associated (IgAV-CA) was defined as IgAV cases where cancer was diagnosed, recurred or was active within 3 years before or after IgAV diagnosis. The definition was adopted from the definition of cancer-associated myositis (17).

We analysed the prevalence, temporal occurrence and characteristics of cancer in IgAV.

### Statistical analysis

The results were expressed as medians and interquartile ranges (IQRs) for non-normally distributed variables, and as means with standard deviations (SD) for normally distributed metric variables. Categorical variables were expressed as absolute numbers and percentages. Fisher’s exact test was used to compare categorical variables, while the Mann–Whitney test was applied for metric variables. The study investigated the role of nine preselected variables for cancer association in IgAV: demographic data (sex, age), smoking status and specific IgAV clinical characteristics (e.g., necrotic purpura, purpura extension, articular, gastrointestinal and renal involvement, and severity of visceral (gastrointestinal or renal) involvement at presentation as a dichotomous variable), using univariate and multivariate logistic regression analysis. A significance threshold of 0.05 was chosen for all analyses.

### Ethics committee approval

The study was approved by the Slovenian National Medical Ethics Committee under number 159/07/13.

## Results

### Setting and patient selection

**Figure 1:**
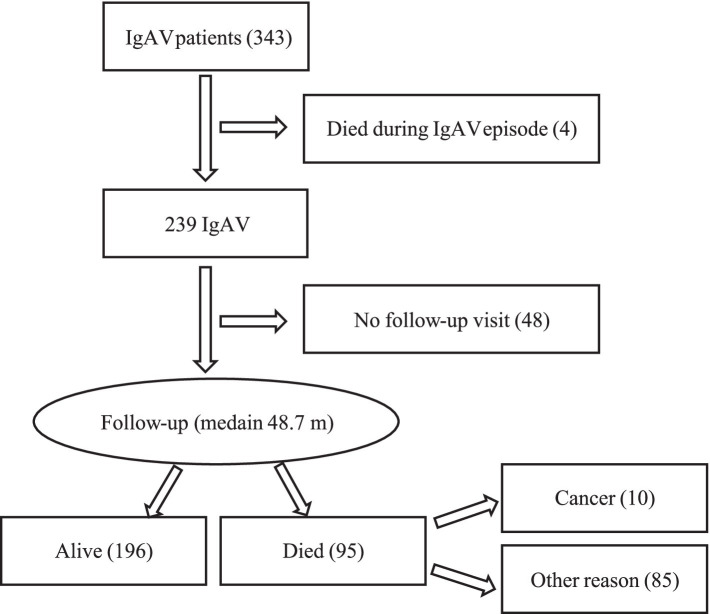
Patient flow chart. IgAV, IgA vasculitis.

Between January 2010 and June 2021, we identified 343 IgAV patients, comprising 206 men (60.1%) with a median (IQR) age of 64.4 (45.4; 76.6) years. Forty-eight patients (14.0%) were lost to follow-up, leaving 295 patients in our study cohort (Figure 1).

### Baseline assessment of IgAV, treatment and follow-up

The characteristics of the 295 IgAV patients are presented in Table 1. Briefly, there were 177 males (60.0%); 132 (44.7%) were ever smokers, and the median (IQR) age at IgAV diagnosis was 64.3 (45.5; 76.2) years. A history of known, past cancer was reported in 38 patients (12.9%).

**Table 1:** Characteristics of IgAV patients: all and those with or without occurrence of cancer within a ±3-year-period from IgAV diagnosis (IgAV-CA vs. non-IgAV-CA).

Characteristics	All IgAV (295)	IgAV-CA^a^ (22)	Non-IgAV-CA (273)	*p* value
Male	177 (60.0%)	17 (77.3%)	160 (58.6%)	0.113
Age^b^	64.3 (45.5; 76.2)	71.4 (64.9; 79.2)	63.4 (44.6; 75.8)	0.013
Ever smoker	132 (44.7%)	13 (59.1%)	119 (47.7%)	0.185
Constitutional sympt.	45 (15.3%)	2 (9.1%)	43 (15.8%)	0.547
Skin necroses	135 (45.8%)	13 (59.1%)	122 (44.7%)	0.266
Purpura above waistline	152 (51.5%)	11 (50.0%)	141 (51.6%)	1.0
Articular involvement	111 (37.6%)	5 (22.7%)	106 (38.8%)	0.171
GIT involvement	85 (28.8%)	5 (22.7%)	80 (29.3%)	0.629
Renal involvement	137 (46.4%)	7 (31.8%)	130 (47.6%)	0.185
Skin limited IgAV	84 (28.5%)	10 (45.5%)	74 (27.1%)	0.085
Severe GIT or renal involvement	54 (18.3%)	4 (18.2%)	50 (18.3%)	1.0
Elevated s-IgA	120/234 (51.3%)	13/18 (72.2%)	107/216 (49.5%)	0.085
BVAS-3^b^	8 (3; 14)	7 (4; 16)	8 (3; 14)	1.0

a Co-occurrence of IgAV and cancer diagnosed / active within a 3-year period.

b Median (IQR).

GIT, gastrointestinal tract; BVAS-3, Birmingham vasculitis activity score-3.

In the clinical presentation of IgA vasculitis, skin involvement was present in all 295 patients (100%), with necrotic purpura in 135 (45.8%). Articular involvement was noted in 111 patients (37.6%), including arthritis in 45 cases (15.3%). Gastrointestinal complications were seen in 85 patients (28.8%), with severe manifestations such as major bleeding or bowel ischemia in 23 patients (7.8%). Renal involvement was observed in 137 patients (46.4%), with severe complications like acute kidney injury or nephrotic syndrome in 36 patients (12.2%). Concurrently with IgAV diagnosis, cancer was newly diagnosed in 3 (1.0%) patients. Additionally, 5 out of 38 patients with a history of past cancer had active or relapsing malignant disease at the time of vasculitis diagnosis.

Treatment of IgAV followed our local practice, as previously described in detail (12), and consisted of systemic glucocorticoids in 211 patients (71.5%). Additional immunomodulation was required in 39 patients (13.2%), with cyclophosphamide used in 27 out of these 39 (69%) patients. Fifteen patients were treated with intravenous immunoglobulins, 3 with plasmapheresis, 2 patients received mycophenolate mofetil, 2 rituximab, 2 dapsone, and 1 patient colchicine. Montelukast was given in 9 cases.

Four patients died due to active vasculitis within the first few days or weeks. The remaining patients were followed for a median (IQR) of 48.7 (18.0; 88.5) months. During follow-up 73 (25.1%) patients had symptoms or signs of persistent IgAV, and 43 patients (14.8%) relapsed. By the censor date for data collection 95 (32.6%) patients died, 10 deaths were cancer related.

During the follow-up period, 9 patients (3.1%) developed a total of 10 new cancers, while 3 patients with a known cancer experienced cancer progression.

### Cancer episode classification

**Figure 2:**
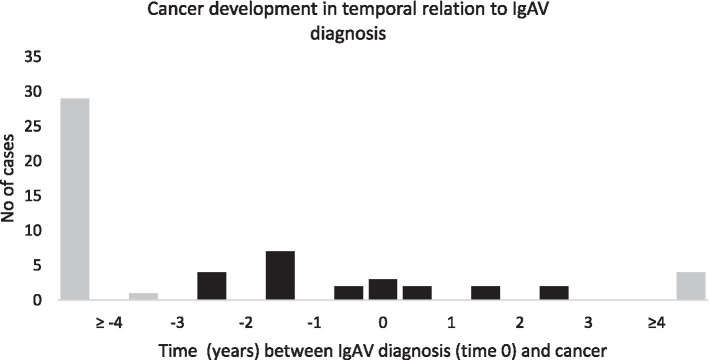
Temporal relation between diagnosis of IgAV and cancer.

A total of 56 different cancers were recorded in 47 patients (15.9%), of which 9 patients developed 2 different cancers. Among these, 43 out of 56 cancers (76.8%) were diagnosed prior to IgAV diagnosis, ranging from 0.7 to 20.5 years prior to diagnosis. Within 3 years prior to IgAV diagnosis, 13 cancers (23.2%) were detected, including 4 cancers that were active or recurrent at the time of IgAV diagnosis. Three cancers (5.4%) in 3 patients were diagnosed concurrently with IgAV, and 10 (17.3%) cancers in 9 patients were diagnosed during IgAV follow-up. Among these 10 cancers (10.7%), 6 were diagnosed within the first 3 years of follow-up. During follow-up, progression or relapse of known cancer was recorded in 3 patients: in 2 patients 30 and 10 years after cancer diagnosis, respectively (11 and 8 years since IgAV diagnosis, respectively); one patient experienced a second recurrence of cancer (with the first recurrence already present at the time of IgAV diagnosis). Figure 2 illustrates the temporal relation between IgAV diagnosis and the first diagnosis of each individual cancer.

**Figure 3:**
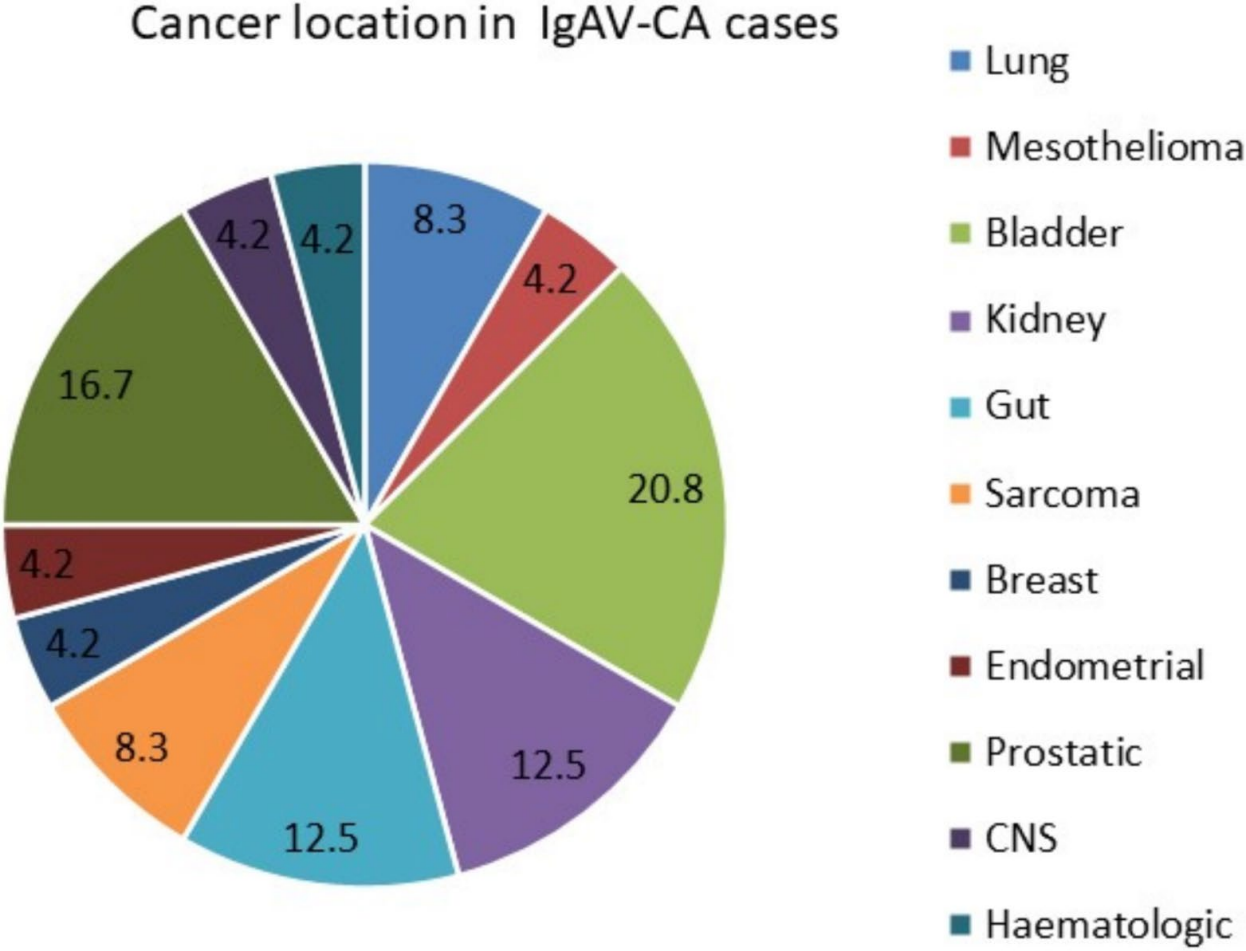
Cancer location in adults with IgAV-CA cases.

Twenty-four different cancers (42.9%) developed or were active within 3 years before or after IgAV diagnosis in a total of 22 patients, who were classified as IgAV-CA cases. Two IgAV-CA patients (1 male and 1 female) developed two different cancers; the rest developed 1 cancer each. In 77.3% of IgAV-CA cases (17 patients), the patients were male, with a median (IQR) age of 71.4 (64.9; 79.2) years (significantly older than the non-IgAV-CA group). Figure 3 shows the location of cancers in the IgAV-CA group. Among the cancers diagnosed, urogenital tract cancers predominated: renal, bladder, prostatic and endometrial cancers together represented 54.2% of cancers in IgAV-CA patients.

In patients who developed 2 different cancers, cancers were localized in bladder and lung (2 cases), and 1 case each of: prostatic cancer and B cell lymphoma; colorectal and thymic cancer, colorectal and bladder cancer, bladder and oral cavity cancer, bladder and prostatic cancer, breast and kidney cancer, Burkitt lymphoma and diffuse large B cell lymphoma.

In IgAV patients who ever developed cancer, as factors associated with cancer emerged increasing age [OR 1.016 (95% CI 1.001; 1.032), *p* = 0.042], and severe visceral IgAV involvement [OR 6.113 (95% CI 3.208; 11.651), *p* < 0.001]. Table 2 shows the results of univariate and multivariate logistic regression analysis. Using logistic regression analysis, we found no associations between the nine evaluated IgAV features and past cancer episodes (prior to IgAV). Similar analysis in patients with cancer being active or diagnosed at the time of IgAV diagnosis revealed an increasing age [OR 1.127 (95% CI 1.031; 1.231), *p* = 0.008] and smoking [OR 11.3 (95% CI 1.675; 76.381), *p* = 0.013] as independent cancer associated risk factors (Table 3). In patients who developed cancer during IgAV follow-up period, arthritis [OR 6.0 (95% CI 1.443; 24.943), *p* = 0.014] emerged as an independent predictor of cancer development (Table 4).

**Table 2:** Associations between IgAV and ever cancer.

Variable	Coefficient	Std. error	*p* value	OR (95%CI)
(a) Univariate logistic regression analysis				
Age	0.023	0.009	0.014	1.023 (1.005; 1.042)
Female gender	−0.010	0.334	0.968	1.014 (0.529; 1.944)
Ever smoking	0.592	0.322	0.066	1.808 (0.962; 3.401)
Articular involv.	0.105	0.336	0.756	1.110 (0.575; 2.145)
Purpura above waistline	0.103	0.318	0.745	1.109 (0.595; 2.067)
Skin necroses	−0.040	0.315	0.899	0.961 (0.518; 1.783)
GIT involv.	0.154	0.370	0.676	1.67 (0.566; 2.408)
Renal involv.	−0.712	0.357	0.046	0.491 (0.244; 0.987)
Severe GIT or renal	2.030	0.407	<0.001	7.616 (3.427; 16.923)
(b) Multivariate logistic regression analysis				
Age	0.016	0.008	0.042	1.016 (1.001; 1.032)
Severe GIT or renal	1.810	0.329	<0.001	6.113 (3.208; 11.651)

GIT, gastrointestinal; OR, odds ratio; CI, confidence interval.

**Table 3:** Associations between IgA vasculitis and concurrent or active cancer.

Variable	Coefficient	Std. error	*p*	OR (95%CI)
(a) Univariate logistic regression analysis				
Age	0.125	0.045	0,006	1.133 (1.037;1.227)
Female gender	−18.846	3103.993	0.995	0
Ever smoking	1.686	1.021	0.098	5.400 (0.730; 39.953)
Arthritis	−18.665	4671.241	0.997	0
Purpura above waistline	0.500	0.887	0.955	1.051 (0.185; 5.978)
Skin necroses	0.77	0.866	0.929	1.080 (0.198; 5.902)
GIT involv.	1.090	1.080	0.313	2.974 (0.358; 24.685)
Renal involv.	−1.611	1.227	0.189	0.200 (0.018; 2.211)
Severe GIT or renal	0.952	1.345	0.479	2.590 (0.185; 36.174)
(b) Multivariate logistic regression analysis				
Age	0.119	0.045	0.008	1.127 (1.031;1.231)
Ever smoking	2.426	0.974	0.013	11.311 (1.675; 76.381)

GIT, gastrointestinal; OR, odds ratio; CI, confidence interval.

**Table 4:** Associations between IgA vasculitis and cancer developing after IgAV.

Variable	Coefficient	Std. error	*p* value	OR (95%CI)
(a) Univariate logistic regression analysis				
Age	0.038	0.026	0.143	1.039 (0.987; 1.094)
Female gender	0.451	0.798	0.572	1.570 (0.329; 7.500)
Ever smoking	0.778	0.857	0.364	2.178 (0.406; 11.680)
Arthritis	2.097	0.815	0.010	8.141 (1.649; 40.204)
Purpura above waistline	0.093	0.797	0.907	1.98 (0.230; 5.232)
Skin necroses	−0.557	0.819	0.497	0.573 (0.115; 2.854)
GIT involv.	−0.586	1.033	0.570	0.556 (0.74; 4.211)
Renal involv.	−0.918	0.897	0.306	0.400 (0.069; 2.318)
Severe GIT or renal	1.155	1.101	0.294	3.173 (0.367; 27.443)
(b) Multivariate logistic regression analysis				
Arthritis	1.792	0.727	0.014	6.0 (1.443; 24.943)

GIT, gastrointestinal; OR, odds ratio; CI, confidence interval.

Older age at IgAV diagnosis, but not IgAV specific characteristics, emerged as a factor associated with IgAV-CA (*p* = 0.006).

## Discussion

Today, autoimmunity and cancer are no longer considered as two separate conditions, but rather as two sides of the same coin (18). Genetic predispositions, environmental factors and epigenetic modifications can lead to immune system dysfunction resulting in autoimmunity or cancer, and common immune pathways are involved in breaking self-tolerance in autoimmunity and inducing tolerance to cancer (19). Moreover, our understanding of the close relation between autoimmunity and cancer has increased with the experiences gained from modern anticancer treatment strategies, such as checkpoint inhibitors and CAR T therapy in which immunotoxicity and autoimmunity are known complications (20, 21). Nevertheless, molecular mechanisms by which cancer can induce vasculitis are still not fully understood. Cancer (neo)antigens can induce immune reaction leading to vasculitis. There may be tumour induced increased autoantibody production, formation of immune complexes and their impaired clearance. Next, immunogenicity might be related to cancer antigens that may share homology to endothelial antigens. A dysregulation of T cells with enhanced IgM to IgA switching has also been described. Cancer cells may release different cytokines that could induce endothelial injury and lead to vessel wall inflammation (22). Finally, various anti-cancer drugs (not only immune check point inhibitors) can induce IgAV—for example through changes in cell surface antigens or increasing the exposure to neoantigens (by killing cancer cells). Data on the association between IgA vasculitis and cancer are relatively scarce.

Our study contributes to the understanding of the association between adult IgAV and cancer. The results of this study show that around 7% of IgAV cases could be associated with cancer when applying the IgAV-CA case definition, which considers active cancer and an IgAV episode occurring within a 3-year window of each other. However, there is currently no widely accepted definition of cancer-associated vasculitis, making direct comparisons of different study results challenging.

A retrospective French study involving 30 IgAV patients with cancer used a different methodological approach to include patients, surveying university and general hospitals. They defined cases where solid cancer was diagnosed within 2 years before or after IgAV diagnosis (13). A large retrospective Spanish study on cutaneous vasculitides reported that around 4% of skin vasculitis were associated with cancer (23). In this study, cancer and vasculitis were considered to be concurrent if both processes were identified within 12 months of each other, with no other known precipitating factor of vasculitis present, and/or a synchronous recurrence of both diseases documented during follow-up (22). Contrary to the Spanish study, our study did not exclude cases with coexisting potential IgAV triggers nor require a synchronous recurrence of both diseases at follow up for inclusion.

Solid cancers significantly predominated in our IgAV-CA patients, representing more than 95% of cancers in this cohort. This finding is in line with a recent literature review that encompassed case reports, case series and rare cohort studies on cancer in IgAV, which reported two-thirds of cancers as solid malignancies (14). This review identified urogenital cancers as the second most frequent cancer, following only lung cancer, but the difference was numerically negligible (24.7% prevalence of lung cancer and 23.3% of urogenital cancers) (14). Interestingly in more than half of our IgAV-CA cases, cancers were also located in the urogenital tract. These results are discordant with those of the aforementioned Spanish study, in which haematological cancers predominated (23).

When evaluating factors associated with ever cancer in our IgAV cohort, it was not surprisingly to find an older age associated with increased risk. As an additional cancer risk factor emerged severe visceral IgAV (either severe gastrointestinal or renal manifestations).Focusing specifically on the IgAV-CA group, only increasing age remained associated with cancer. Likewise, in the French cohort, IgAV patients with cancer were older compared to a control IgAV group. Hankard et al. also reported that patients with cancer have a more severe IgAV phenotype with more frequently necrotic purpura and intra-alveolar hemorrhage (13). However, their conclusions were not replicated by our study, possibly due to different study design and patient recruitment.

While we did not find any association between history of past cancer and the clinical presentation of IgAV, we observed an increasing age and ever smoking as factors associated with cancer active/concurrent to IgAV diagnosis. In addition, arthritis emerged as a risk factor for cancer development during IgAV follow-up. Contrary to our results, Mitsui et al. found arthralgias more frequently in the IgAV group without associated cancer than in cancer associated group, but the association between older age and cancer was present also in their cohort. Interestingly they observed daily proteinuria (>1 g) more commonly in noncancer compared to cancer IgAV patients (15).

The design of our study was not aimed to provide cause-and-effect relation between IgAV and the development of cancer. Further limitations include its single-centre design and a relatively small number of events, despite the extended duration of patient inclusion and the significant number of IgAV cases included. The study clearly demonstrates how relatively rare events require long-term follow-up and a large number of participants. In future prospective studies with similar research questions, incorporating modern whole-body imaging (e.g., PET scans) would be interesting to consider as a way to increase the reliability of the study results. However, practical considerations such as time, costs, and ethical constraints limit the feasibility of such approaches in routine clinical practice. In addition, family history of cancer and autoimmune disease was not assessed in this study.

We believe the study also has several strengths. In addition to being prospective, our patients represented an unselected patient population with varying degrees of IgAV severity, including both in-and out-patient managed cases. Furthermore, our rheumatology unit serves as the only secondary referral centre for a population of around 720,000 adults and provides tertiary-level care to around three-quarters of the country’s population. This setup has allowed us to largely avoid any selection bias. In the available literature, cancer reports are often limited to case reports and small case series.

In summary, our study demonstrates that cancer-associated IgAV cases account for around 7% of IgAV cases in adults. Furthermore, our findings underscore the importance of considering cancer screening, particularly focusing on the urogenital tract, when managing elderly patients diagnosed with IgAV.

## Data Availability

The raw data supporting the conclusions of this article will be made available by the authors, without undue reservation.
